# Study protocol for ‘PROMIsE’: Implementation of a curriculum to stimulate PROactive behavior in MIdwifery Education

**DOI:** 10.18332/ejm/94653

**Published:** 2018-09-04

**Authors:** Eveline Mestdagh, Bart Van Rompaey, Olaf Timmermans

**Affiliations:** 1 Midwifery Department, Artesis Plantijn University College Antwerp, Antwerpen, Belgium; 2 Centre for research and innovation in care, University Antwerp, Belgium; 3 Lectoraat Healthy Region, HZ University of Applied Sciences, Vlissingen, The Netherlands

**Keywords:** midwifery students, proactive behavior, role breadth selfefficacy, autonomy, control appraisal, trust in peers

## Abstract

**INTRODUCTION:**

Proactive behavior shows promise in the challenges of midwifery students in adapting quickly and effectively to different clinical settings. The antecedents of rolebreadth self-efficacy, control appraisal and trust in peers have demonstrated a potential for significant benefit to proactive behavior in midwifery education. A new midwifery educational program, ‘PROMIsE’, was developed to influence these antecedents and so enhance proactive behavior.

**METHODS:**

A pre-test/post-test cohort study of midwifery students’ antecedents in proactive behavior will be conducted from September 2018 until June 2022. All new starting midwifery students (n = estimated at 150) at one Belgian University College will be included. Data will be collected using a validated questionnaire at four time points: the entry point in the new midwifery curriculum, after one year, two years and at the end of the curriculum. A proportional odds logistic regression analysis will be used to clarify the association between these antecedents and the probability to observe proactive behavior within this group at different time points.

**RESULTS:**

A historical comparison will be made with this cohort study and two previous cross-sectional studies. With ‘PROMIsE’ it is assumed that this cohort, which underwent the intervention of ‘PROMIsE’, will score significantly higher than the cross-sectional study groups.

**CONCLUSIONS:**

‘PROMIsE’ aims to support the individual guidance of midwifery students towards proactive behavior in midwifery in order to cope with the numerous challenges in reproductive healthcare.

## INTRODUCTION

Proactive behavior in midwifery could bring benefits to the constantly evolving field of reproductive health care^1^. Midwives behaving proactively, are always one step ahead, adapt easily, work autonomously and are constantly in search for the most effective and qualitative state-of-the-art care provision. Due to the continuing focus on improving quality and work-efficiency, proactive behavior has been identified as an important coping mechanism for managing stress^2, 3^. Moreover, increased proactive behavior is related to higher job satisfaction, a stronger commitment, improved productivity of the midwifery team and, therefore, is a determining factor for organizational success^4,5,6,7^. To promote and strengthen proactive behavior in midwifery, as early as possible, intervening in midwifery education is considered the logical next step.

### Background

Current debate in midwifery education focuses on how to educate midwives in the 21st century, as new or expanded roles and skills for midwives are essential for a higher quality of healthcare and for the development of the profession^8^. Educational programs should not focus solely on practical skills, but also on attitudinal competences and behavioural development, to stimulate quick and viable coping in the constantly evolving continuity of midwifery care^9^. Education plays a key role in helping students develop resilience, providing them with the skills to handle both the workplace environment and personal stress^10^.

Drawing on the literature, proactive behavior of the midwife is identified as adding significant value to coping with the challenges of working in the continuously evolving reproductive health care. Proactive behavior is a universal concept that can be generalized to many professions. Initially, this research team attempted to disentangle and theoretically apply the concept of proactive behavior within midwifery. By means of a concept analysis, according to the method of Walker and Avant^11^, eight steps were taken to understand the evolution of the concept of proactive behavior in midwifery and to make a clear distinction between relevant and irrelevant characteristics^1^. Based on this theoretical study, a number of personal and contextrelated prognostic antecedents emerged, possibly linked to whether or not proactive behavior occurred. All the antecedents, stemming from the concept analysis, supported the compilation of a questionnaire to determine the influence of individual and/or contextual antecedents; for example, job autonomy and control appraisal towards proactive behavior. This questionnaire was first tested in a cross-sectional pilot-study group of midwifery students (n = 156) in the academic year 2015-2016^10^. Afterwards, the questionnaire was re-plotted and validated in a follow-on cross-sectional study, as described in chapter 4, wherein a larger sample of midwifery students (n = 421) in Flanders were questioned in the academic year 2017-2018^12^. According to both crosssectional studies, midwifery students who have a high-role breadth self-efficacy, referring to the self-confidence of a midwifery student to perform tasks that exceed expectations, a higher number of years into the study program, a high trust in their peers and low control appraisal are more likely to show proactive behavior. Additionally, students with the Dutch nationality were more likely to show proactive behavior, compared to Belgian students. The low score on control appraisal suggests that midwifery students who have the tendency to show proactive behavior do not immediately feel the importance attached to one’s perceived control.

Results of both studies were captured and added into a newly developed educational program, called ‘PROMIsE’, for the Bachelor degree in midwifery at a Belgian University College. This program commenced for the first time in September 2017. The research team took the first year of the educational program to monitor possible shortcomings and/or add extra tools. This study protocol seeks to evaluate the effect of ‘PROMIsE’ on the antecedents associated with proactive behavior.

## METHODS

### Research hypothesis

This study protocol hypothetically states that midwifery students, undergoing ‘PROMIsE’, will have a higher, rolebreadth self-efficacy and trust in peers and a lower level of control appraisal, and eventually will behave more proactively in practice.

### Study design

**Figure 1:**
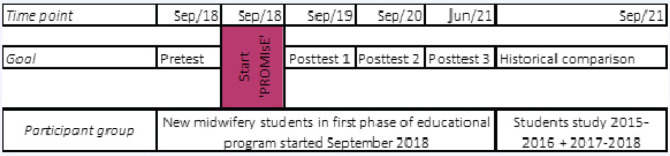
Study timeline

This study will employ a pre-test/post-test approach by use of the validated questionnaire of Mestdagh et al.^13^ and will consist of two phases to explore the level of proactive behavior at four time points (Figure 1).

**Figure 2:**
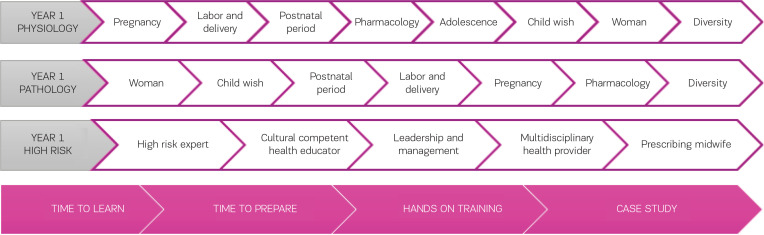
Life stages of the study program of the Midwifery Department of the participating University College

Ensuring adherence to study protocol-reporting standards, the SPIRIT 2013 checklist was followed.

#### Phase 1

##### Time 1

All new midwifery students at the participating University College will be asked to complete the validated questionnaire at their entry point in September 2018. This phase is considered the baseline assessment.

##### Time 2-3-4

The same group of students will be asked to fill in the questionnaire after the first, second and, at the end of their midwifery study program, the 3rd year in June 2021.

#### Phase 2

A historical comparison will be made of the intervention group and both studied groups in the original pilot^13^ study of 2016 and the validating group^12^ of 2018.

### Intervention

‘PROMIsE’ is a new educational program developed at the Midwifery Department of the participating University College in order to promote proactive behavior of midwifery students. The program is based on four keystones:

#### Keystone 1: Transparent and accessible

To stimulate students’ personal interest and intrinsic motivation, a transparent educational program has been developed, structured into different life stages in which the midwife has a crucial role. The explicit naming of the life stages will help the (future) midwife to understand the focus of that section of the study program.

Based on the four components/instructional design (4C/ ID)-model of Merriënboer^14^, authentic learning tasks have been developed. Such tasks are instrumental in helping student midwives to integrate knowledge, skills and attitudes (competences).

Each ‘stage of life’ will take one or more weeks. Within each stage, a clearly communicated, fixed structure is used. Each Monday and Tuesday theoretical classes, called ‘time to learn’, will be organised. On Wednesdays ‘time to prepare’ will be provided, in which the students can process the theory of that week and prepare themselves for the next two days. Thursdays, specific practical skills related to the specific life stage, will be exercised in small groups in the ‘hands-ontraining-sessions’. Driven by the case-based learning system, each Friday students will be guided in the ‘case study groups’ where they will integrate the acquired knowledge through case discussions in smaller groups under the supervision of teachers and (clinical) midwives.

#### Keystone 2: Evidence based

‘PROMIsE’ cooperates in various research projects within both the midwifery and educational areas. Hereby, an attempt is made to create a continuous cross-fertilization of scientific results and day-to-day learning and practice. The students are encouraged to adopt a critical research attitude. They are challenged to underpin each case study critically, using actual scientific literature and evidence.

#### Keystone 3: Focus on workplace learning

In a safe and simulated in-house skills’ laboratory environment, basic technical, obstetric and neonatal skills, based on authentic professional situations, will be taught. In this way, students will be properly prepared for clinical practice. The number of clinical placements have gradually increased each year. All students go through all midwifery domains, as described in the European Directives, both national and international^15^. The final test for each student is involvement in a complex internship structure, where a group of final year students literally is in charge of the complete midwifery unit for a minimum of six weeks.

#### Keystone 4: Proactively focused on society

All relevant stakeholders, e.g. political partners, clinical midwives, students, will regularly meet to discuss and evaluate the content, processes and evaluation methods of ‘PROMIsE’. Current expertise, by means of guest lecturers, are actively included in the program. All involved midwifery lecturers are very active members in both national and international professional organizations related to midwifery. In this way, ‘PROMIsE’ attempts to have a constant grip on the actual and social trends and movements in midwifery. With very little use of static learning material and by means of a very active digital learning environment, ‘PROMIsE’ will quickly respond to the newest trends and evidence. It is assumed that midwives who graduate from the participating University College will be better qualified for the needs of the labor market and society.

### Sampling procedures and study participants

#### Intervention group

All new registered student midwives, who will start the educational program in September 2018, will be approached through the digital learning platform by the head of midwifery education, who is one of the authors of this study protocol. A total of 150 students is expected.

#### Control groups

1. The 156 midwifery students who studied at the same University College before the introduction of ‘PROMIsE’ and participated in the cross-sectional pilot study^16^ of 2015–2016.
2. The 421 midwifery students who studied in one of the nine educational midwifery programs in Flanders before the introduction of ‘PROMIsE’ and participated in the cross-sectional validation study^12^ of 2017–2018.

### Ethical considerations

The study was approved by the management and research director of midwifery education at the participating University College. The ethical commission of social and human sciences at the University of Antwerp, Belgium (SHW_17_48_02) gave ethical approval. An informed consent form, with information about the aim and design of the study will be given to the participants. All will be referred to the confidential aspect of their participation and reassured that they would not be identifiable in any reports or published work.

### Validity and reliability

Three recent studies validated the questionnaire to assess proactive behavior in midwifery students^12,13,16^. Based on the results of these three previous studies and the content validity check, both on the item (I-CVI, 0.90–1.00) and scale (S-CVI, 0.89–1.00) level, a 65-item questionnaire with a set of seven individual and three contextual antecedents related to proactive behavior was developed (Appendix 1). All antecedents are assessed using existing and validated tools inspired by the study of Parker et al.^17^. A blank space will be added to the questionnaire to allow participants to add helping and/or hindering factors they have experienced with ‘PROMIsE’ in daily practice.

For the historical comparison purpose, all measured antecedents will be centered around the median value used in our previous study^13^.

### Data collection and analysis

All students will be asked to fill in the validated questionnaire at the predetermined time points. Questionnaires will be coded and processed anonymously by the research team. The completion of the questionnaire is estimated to take on average 15 minutes. The raw data from each student’s responses will be coded numerically. Questions where multiple answers are possible will be coded: 4 for totally agree, 3 for agree, 2 for disagree and 1 for totally disagree. Summedscores per student per antecedent will be used in the analysis.

To describe the distribution of proactive behavior in these study populations, proportional odds logistic regression will be used. Proportional odds models are necessary in this case, as not only the categorical nature of proactive behavior is taken into account but also the ordinal relationship between the levels of proactive behavior. Fitting of the models to the observed data, and subsequent evaluation of the model’s goodness-of-fit and the uncertainty of the estimated parameters, will be performed in R^©^.

## DISCUSSION

This study focuses on the effect that ‘PROMIsE’ will have on the level of proactive behavior of midwifery students. The research team believes that ‘PROMIsE’ will affect the rolebreadth self-efficacy, control appraisal and trust in peers of the midwifery students. Attention for proactive behavior can provide resilience and support for (future) midwives, so that (future) mothers and fathers will be able to deal with social changes quickly.

First, the blended learning package, combining different learning methodologies, stimulates self-regulation that in itself promotes reflection^18^. A high level of reflection skills is decisive for proactive behavior^19^. Second, team-based assignments in each case study-group could foster the development of students’ teamwork skills and therefore increase the students’ trust in peers^20^. Third, the clear and transparent competence-based structure of the program enhances the feeling of being in control, and therefore lowers the need for control appraisal^21,22^. When students experience that their learning environment is controllable on matters of import to them, they are motivated to fully exercise their personal efficacy, which enhances their likelihood of success. In turn, experiences of success are directly related to the development of role breadth self-efficacy^23^. Fourth, increased task control and a feeling of membership are positively associated with increased role breadth self-efficacy^24^. In addition, communication has been proven to promote role breadth self-efficacy. The more students feel that they are informed, listened to, and encouraged, the more likely they will develop confidence in carrying out a range of proactive, interpersonal, and integrative tasks.

### Limitations

First, a limitation of ‘PROMIsE’ is that some components of the study program, such as pharmacology, are organized as separate study modules due to legal obligations, which could hinder the overall integration. Second, although ‘PROMIsE’ is transferable to other international midwifery programs, some outcomes might be typical of the Flemish midwifery educational program. Since Flemish midwifery departments are not allowed to impose conditions on the enrolment of students, it could provide a distorted picture in programs that thoroughly select students before the start of the study. A not insignificant prerequisite for the success of ‘PROMIsE’ is the safe and supportive culture of the educational department within which the midwifery student can increase her/his level of willingness to behave proactively^25^. As Edmondson et al.^26^ stated, psychological safety, being the shared belief that safety is guaranteed for interpersonal risk taking, could be a stimulating factor for team learning and/or innovation. Also, the informal belief and motivation of the student that proactive behavior will bring about a set of salient outcomes, are important determinants of the midwifery student’s attitude^27,28^.

Midwifery education and practice primarily focus upon the physical and mental care for women. Additional advanced professional development skills, such as proactive behavior, are much less delineated. Sharing ‘PROMIsE’ with other international midwives, could be the next step in the ongoing search for understanding and promoting proactive behavior in midwifery education and practice.

## CONCLUSIONS

This study seeks to evaluate the effectiveness of ‘PROMIsE’ on the level of proactive behavior by equipping midwifery students with higher role breadth self-efficacy, autonomy, trust in peers and a lower need for control appraisal. If positive results are yielded, future studies can explore the sustainability and expansion of the program to more midwifery departments in other university colleges. Ultimately, this study protocol is the first step in providing an evidencebased intervention to successfully instill proactive behavior in future midwives.

## CONFLICTS OF INTEREST

Authors have completed and submitted the ICMJE Form for Disclosure of Potential Conflicts of Interest and none was reported.
